# Aerobic Interval Training Attenuates Mitochondrial Dysfunction in Rats Post-Myocardial Infarction: Roles of Mitochondrial Network Dynamics

**DOI:** 10.3390/ijms15045304

**Published:** 2014-03-26

**Authors:** Hong-Ke Jiang, You-Hua Wang, Lei Sun, Xi He, Mei Zhao, Zhi-Hui Feng, Xiao-Jiang Yu, Wei-Jin Zang

**Affiliations:** 1 Department of Pharmacology, College of Medicine, Xi’an Jiaotong University, Xi’an 710061, China; E-Mails: jianghk88@126.com (H.-K.J.); wangyh26@126.com (Y.-H.W.); xjtusunlei@126.com (L.S.); hexi1818@163.com (X.H.); zmxjtu@126.com (M.Z.); yxs@mail.xjtu.edu.cn (X.-J.Y.); 2 Department of Physical Education of Nan Yang Institute of Technology, Nanyang 473000, China; 3 Center for Mitochondrial Biology and Medicine, Key Laboratory of Biomedical Information Engineering of the Ministry of Education, School of Life Science and Technology, Frontier Institute of Science and Technology, Xi’an Jiaotong University, Xi’an 710049, China; E-Mail: zhfeng@sibs.ac.cn

**Keywords:** myocardial infarction, mitochondrial dysfunction, fusion, fission, mitochondrial dynamics, aerobic interval training

## Abstract

Aerobic interval training (AIT) can favorably affect cardiovascular diseases. However, the effects of AIT on post-myocardial infarction (MI)—associated mitochondrial dysfunctions remain unclear. In this study, we investigated the protective effects of AIT on myocardial mitochondria in post-MI rats by focusing on mitochondrial dynamics (fusion and fission). Mitochondrial respiratory functions (as measured by the respiratory control ratio (RCR) and the ratio of ADP to oxygen consumption (P/O)); complex activities; dynamic proteins (mitofusin (mfn) 1/2, type 1 optic atrophy (OPA1) and dynamin-related protein1 (DRP1)); nuclear peroxisome proliferator-activated receptor gamma coactivator 1-alpha (PGC-1α); and the oxidative signaling of extracellular signal-regulated kinase (ERK) 1/2, c-Jun NH_2_-terminal protein kinase (JNK) and P53 were observed. Post-MI rats exhibited mitochondrial dysfunction and adverse mitochondrial network dynamics (reduced fusion and increased fission), which was associated with activated ERK1/2-JNK-P53 signaling and decreased nuclear PGC-1α. After AIT, MI-associated mitochondrial dysfunction was improved (elevated RCR and P/O and enhanced complex I, III and IV activities); in addition, increased fusion (mfn2 and OPA1), decreased fission (DRP1), elevated nuclear PGC-1α and inactivation of the ERK1/2-JNK-P53 signaling were observed. These data demonstrate that AIT may restore the post-MI mitochondrial function by inhibiting dynamics pathological remodeling, which may be associated with inactivation of ERK1/2-JNK-P53 signaling and increase in nuclear PGC-1α expression.

## 1. Introduction

Acute myocardial infarction (MI) is a chief cause of morbidity and mortality, with increasing global prevalence [1]. After MI, the remodeled heart is characterized by mitochondrial dysfunction, and the importance of mitochondrial abnormalities in the progression of chronic heart failure (CHF) is broadly accepted [2–4]. A compelling body of evidence links mitochondrial dysfunction to MI and/or CHF by demonstrating defects in mitochondrial respiratory function and decreased respiratory marker enzyme activities in these conditions [5–7]. Mitochondria are dynamic organelles that can change their morphology by undergoing continuous fusion and fission, which generates an elongated network or a fragmented discrete phenotype. In mammals, the mitochondrial outer membrane is controlled by fusion-associated proteins (mitofusins; mfns) 1 and 2; the inner membrane is governed by the conserved protein optic atrophy (OPA) 1; and fission is driven by dynamin-related protein (DRP) 1 [8]. Recently, a strong involvement of mitochondrial network morphology, dynamic proteins, and energy metabolism has been reported [9,10]; thus, balanced fusion-fission and a stable protein quality control system are essential for mitochondrial structure and function. Therefore, mitochondrial dynamic proteins may be promising therapeutic targets for treating MI-caused mitochondrial dysfunction.

Endurance training may exert salutary effects on MI-induced mitochondrial dysfunction [11–13] and is a valuable and effective modality for improving the adaption of mitochondrial dynamics in skeletal muscle [14,15]. Presumably, endurance training-induced mitochondrial dynamics remodeling may also have favorable effects on MI-caused mitochondrial abnormalities. However, the impacts and/or mechanisms of endurance training on mitochondrial dynamics in the post-MI myocardium are still unexplored. Constant repeated exercise has a general protective effect against cardiovascular diseases; however, some disadvantages of repetitive exercise, such as single modes of action, lack of rest, or high time input, could reduce practitioner enthusiasm and prevent individuals with little spare time from exercising. In practice, the use of repetitive exercises as a recovery program for MI patients is still controversial. In recent years, aerobic interval training (AIT), which is an endurance training method borrowed from athletic training programs and consists of a higher intensity workload followed by lower intensity recovery [16], has received growing attention [17–19]. In contrast to traditional modalities, the AIT alternative action model may increase interest and provide appropriate rest for practitioners, which is essential for maintaining exercise motivation and achieving the training objectives. AIT, performed with treadmill training or bicycling, induces superior effects with less exercise volume or time investment for patients who have suffered from chronic heart failure [16,20], MI [21], hypertension [22] and coronary heart disease [23], indicating that AIT is characterized by time-efficiency. Therefore, AIT may be a better choice than traditional exercise methods for heart failure patients who are either in clinical treatment or in a home-based cardiac rehabilitation program.

Although AIT promotes the recovery of pathological myocardium [24], experimental evidence describing how AIT affects mitochondrial network proteins and functional performance in failing cardiac muscle remains scarce. Moreover, AIT reportedly increases the expression of peroxisome proliferator-activated receptor gamma coactivator 1-alpha (PGC-1α), which beneficially affects mitochondrial network dynamics [20,24], but one recent study indicated that nuclear PGC-1α expression was more accurate (than total levels) for reflecting the state of the PGC-1α [25]. The specific benefits and mechanisms of AIT to nuclear PGC-1α, especially in peri-infarcted regions (which are characterized by insufficient blood perfusion and will die without intervention), remain unclear. These unresolved questions hamper the mechanistic understanding of new and improved options (AIT) for the prevention of MI-induced adverse events.

Therefore, this study was performed to evaluate the key roles of ameliorating mitochondrial dynamic protein expression in AIT-mediated mitochondrial protection in post-infarct rat ischemic myocardium. Mitochondrial membrane potential and functional performance, including respiration, energy synthesis and complex activities, were assayed. To corroborate this hypothesis, we focused on mitochondrial dynamics networks (fusion and fission). Mitochondrial dynamics-associated regulatory signal cascades were also evaluated.

## 2. Results

### 2.1. Effects of MI and AIT on Left Ventricular Tissue Microstructure

**Figure 1.**
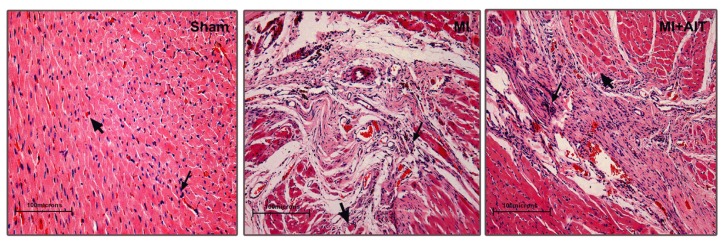
Microstructural changes of left ventricular tissue after MI and AIT intervention. After 8 weeks, the rats were killed, and left ventricular tissue microstructure was determined with HE. 
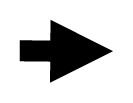
 indicates nuclei (blue); 
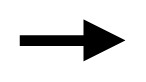
 indicates cytoplasm (pink). Sham: sham-operation; MI: myocardial infarction; MI + AIT: myocardial infarcted rats subjected to aerobic interval training. Bar: 100 μm. *n* = 6 per group.

To verify the hypothesis that AIT can exert beneficial effects on the post-MI heart, left ventricular tissue microstructure was first assessed using hematoxylin-eosin (HE) staining. As demonstrated in Figure 1, normalized morphology, clear boundaries for neatly arranged myocardial cells, and plump muscle bundles and myocardial fibers were observed in the sham group. After MI, typical pathological modification was noted as demonstrated by swelled cardiomyocytes, condensed and fragmented nuclei, broken myocardial fibers, dissolved stripes and disordered myocardial fibers. These detrimental changes were attenuated by AIT, as evidenced by decreases in ruptured myocardial fibers and in disarranged and dissolved stripes.

### 2.2. Effects of MI and AIT on Membrane Potential and Cytochrome C Leakage

**Figure 2.**
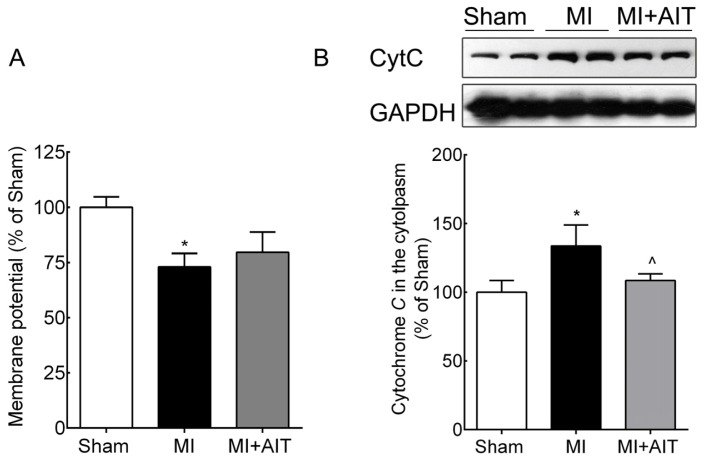
Changes in mitochondrial membrane potential and cytochrome C leakage after MI and AIT intervention. After 8 weeks, the rats were killed, and mitochondrial membrane potential and cytoplasmic cytochrome C levels were assessed. (**A**) Mitochondrial membrane potential; (**B**) cytochrome C (Top: Western blot images; bottom: statistical results). The significance of the differences was calculated using one-way ANOVA followed by Tukey’s *post-hoc* test. The data are presented as the means ± SEM (*n* = 10 for membrane potential; *n* = 8 for cytoplasmic cytochrome C levels). * *p* < 0.05 *vs.* the sham group, ^ *p* < 0.05 *vs.* the MI group. Sham: sham operation; MI: myocardial infarction; MI + AIT: myocardial infarcted rats subjected to aerobic interval training.

Lower mitochondrial membrane potential and increased cytochrome C release were two essential indicators of mitochondrial injury [26], we next evaluated the mitochondrial membrane potential and the protein levels of cytochrome C in each group. As demonstrated in Figure 2A, MI decreased the mitochondrial membrane potential (~30%, *p* < 0.05) compared with the sham group. Although AIT elevated the membrane potential, no significant difference was observed compared with the MI group (*p* > 0.05). Figure 2B also demonstrated increased cytoplasmic cytochrome C content after MI (~30%, *p* < 0.05), indicating that MI damaged mitochondria and increased cytochrome C release. AIT effectively attenuated cytochrome C leakage (*p* < 0.05).

### 2.3. Effects of MI and AIT on Mitochondrial Respiratory Functions, Electron Transport Chain (ETC) Complex Activities and Protein Expression of Citrate Synthase (CS)

**Figure 3.**
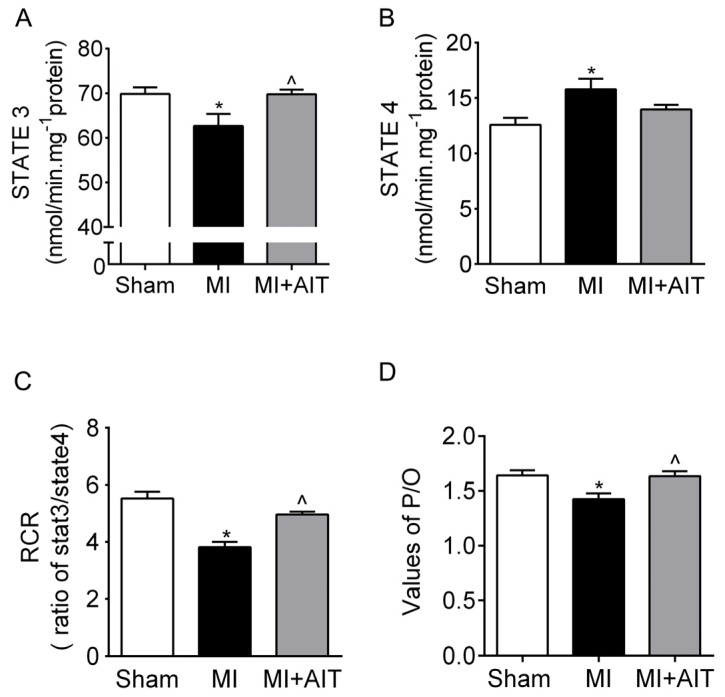
Effects of MI and AIT on mitochondrial respiratory function. After 8 weeks, the rats were killed, and mitochondrial respiration functions were evaluated. (**A**) State 3 of cardiac mitochondrial respiration; (**B**) State 4 of cardiac mitochondrial respiration; (**C**) Cardiac mitochondrial respiratory control ratio (RCR = State 3/State 4); and (**D**) P/O ratio for each group. The significance of the differences was calculated using one-way ANOVA followed by Tukey’s *post-hoc* test. The data are presented as the means ± SEM (*n* = 10). * *p* < 0.05 *vs.* the sham group, ^ *p* < 0.05 *vs.* the MI group. Sham: sham operation; MI: myocardial infarction; MI + AIT: myocardial infarcted rats subjected to aerobic interval training.

Alleviated mitochondrial injury is closely related to functional improvements. We next evaluated mitochondrial respiratory function in samples from the groups. As demonstrated in Figure 3A–C, the State 3 value in the MI group, which is the respiratory rate stimulated by ADP exposure, was lower than that of the sham group (*p* < 0.05). However, during State 4, which represents basal respiration, the MI group values were increased by 20% compared with the sham group. Consequently, the respiration control ratio (RCR, State 3/State 4) was lower than in the sham group (~25%, *p* < 0.05). However, the respiratory function (State 3 and RCR) of the MI + AIT group demonstrated improvements (~20% and 18%, *p* < 0.05). The ratio of ADP to oxygen consumption (P/O ratio) was also markedly decreased by MI (*p* < 0.05) and was moderately restored by AIT (Figure 3D) (*p* < 0.05), which reflects ATP synthesis efficiency.

**Figure 4.**
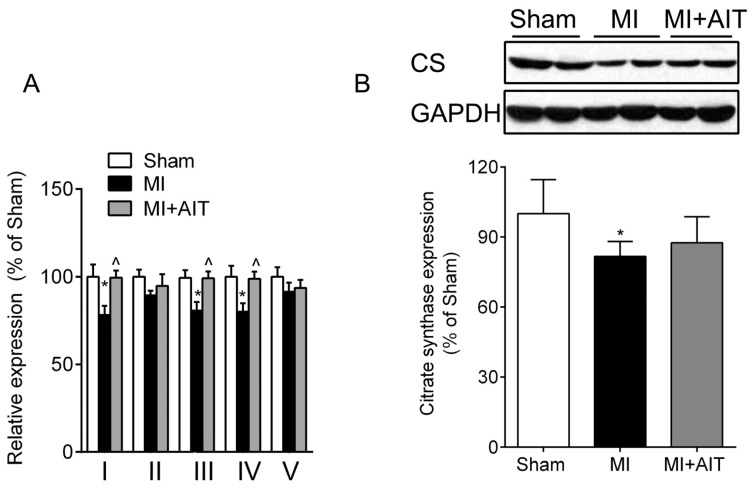
Effects of MI and AIT on mitochondrial electron transport chain (ETC) complex activities and citrate synthase. After 8 weeks, the rats were killed, and ETC complex activities and citrate synthase were assessed. (**A**) ETC complex activities; (**B**) Citrate synthase (CS) expression (Top: Western blot images; bottom: statistical results). The significance was calculated using one-way ANOVA followed by Tukey’s *post-hoc* test. The data are presented as the means ± SEM (complex’s activities, *n* = 10; citrate synthase, *n* = 8). * *p* < 0.05 *vs.* the sham group, ^ *p* < 0.05 *vs.* the MI group. Sham: sham operation; MI: myocardial infarction; MI + AIT: myocardial infarcted rats subjected to aerobic interval training.

If AIT can lessen MI-induced mitochondrial damage, then more preserved mitochondria and improved complex activities are expected in the MI + AIT group. As demonstrated in Figure 4A, after MI, complex I–V activities were lower than those from the sham group. AIT enhanced complex I, III and IV (*p* < 0.05) but not complex II and V (*p* > 0.05) activities. In addition, CS, one rate-limiting enzyme of the Krebs cycle and located in the mitochondrial matrix, is usually found to reflect functional alterations of the mitochondria. We next determined CS modifications in each group animals. Compared with the Sham group, CS expression in the post-MI animals was significantly decreased (*p* < 0.05). AIT failed to significantly increase the protein expression of CS (*p* > 0.05) compared with that of MI sedentary animals.

### 2.4. Effects of MI and AIT on Remodeling of Mitochondrial Dynamics and Oxidative Signaling

**Figure 5.**
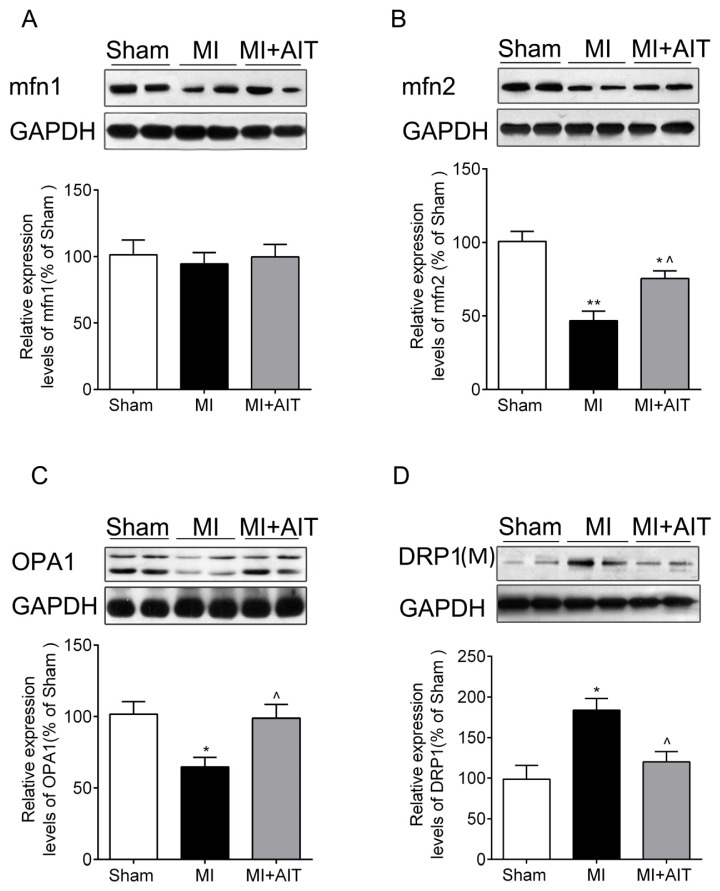
Effects of MI and AIT on mitochondrial dynamic proteins. After 8 weeks, the rats were killed, and the expression levels of mitochondrial proteins mfn1, mfn2, OPA1, and DRP1 were determined by Western blot. (**A**) mfn1; (**B**) mfn2; (**C**) OPA1; (**D**) DRP1. (Top: Western blot images; bottom: statistical results). The significance was calculated using one-way ANOVA followed by Tukey’s *post-hoc* test. The data are presented as the means ± SEM (*n* = 8). * *p* < 0.05 and ** *p* < 0.01 *vs.* sham group; ^ *p* < 0.05 *vs.* MI group; M: mitochondria; Sham: sham operation; MI: myocardial infarction; MI + AIT: myocardial infarcted rats subjected to aerobic interval training.

Mitochondria are highly dynamic organelles and are characterized by dynamic changes in their tubular structures or networks through fusion and/or fission by dynamic protein regulation [9]. We assessed mitochondrial fusion and fission protein expression. As demonstrated in Figure 5B,C, after MI, mfn2 and OPA1 levels were significantly lower than in the sham group (*p* < 0.05); however, DRP1 levels (Figure 5D) were higher (*p* < 0.05). Although mfn1 levels were also decreased, this decrease failed to reach significance (*p* > 0.05) (Figure 5A). AIT increased mfn2 and OPA1 expression (*p* < 0.05) and downregulated DRP1 levels (*p* < 0.05) (Figure 5B–D).

**Figure 6.**
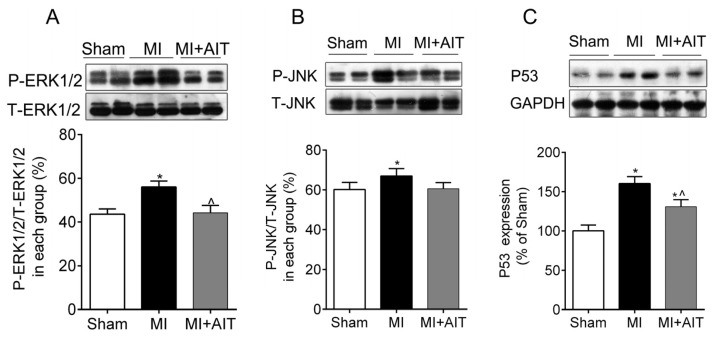
Effects of MI and AIT on oxidative signaling pathway. After 8 weeks, the rats were killed, and oxidative signaling protein expression was evaluated. The expression of oxidative signaling pathway ERK1/2-JNK-P53 was assessed by Western blot. (**A**) P-ERK1/2/T-ERK1/2; (**B**) P-JNK/T-JNK; (**C**) P53 expression (Top: Western blot; bottom: statistical results). Significance was calculated using one-way ANOVA followed by Tukey’s *post-hoc* test. The data are presented as the means ± SEM (*n* = 8 for oxidative pathways). * *p* < 0.05 *vs.* the sham group; ^ *p* < 0.05 *vs.* the MI group. Sham: sham operation; MI: myocardial infarction; MI + AIT: myocardial infarcted rats subjected to aerobic interval training.

Cheng *et al*. recently showed that DRP1 is regulated by P53, which is an important downstream effector of the extracellular signal-regulated kinases (ERK)1/2 and c-Jun *N*-terminal protein kinase (JNK)1/2 oxidative pathway [27]. Presumably, MI-induced increases in mitochondrial fission are associated with oxidative signaling activation. Next, oxidative signaling was evaluated. As demonstrated in Figure 6A, after MI, the ERK1/2-JNK oxidative pathway was activated (*p* < 0.05), and P53 levels were significantly increased (*p* < 0.05). In comparison, AIT inactivated of the ERK1/2-JNK-P53 signaling cascade.

### 2.5. Effects of MI and AIT on PGC-1α and Tfam

**Figure 7.**
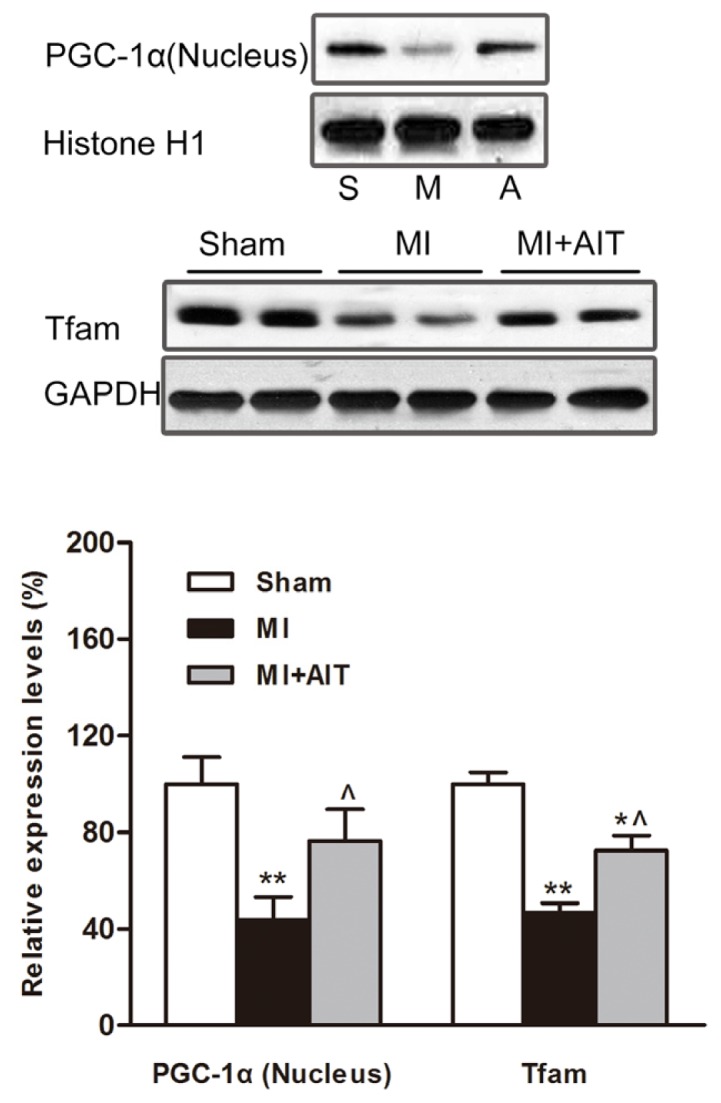
Effects of MI and AIT on PGC-1α and Tfam. After eight weeks, the rats were killed, and the expression levels of nuclear PGC-1α and Tfam were determined by Western blot (Top: Western blot images; bottom: statistical results). Significant differences were calculated using one-way ANOVA followed by Tukey’s *post-hoc* test. The data are presented as the means ± SEM (*n* = 8). * *p* < 0.05 and ** *p* < 0.01 *vs.* sham group, ^ *p* < 0.05 *vs.* MI group. Sham (S): sham operation; MI (M): myocardial infarction; MI + AIT (A): myocardial infarcted rats subjected to aerobic interval training.

In addition to the oxidative stress signal cascade, recent studies indicate that PGC-1α, especially for the nuclear PGC-1α, may be a key regulator of mitochondrial dynamic proteins [28]. In addition, mitochondrial transcription factor A (Tfam) is an important effecter of PGC-1α. Therefore, we evaluated the nuclear PGC-1α protein expression and Tfam expression. As demonstrated in Figure 7, MI downregulated Tfam protein levels (*p* < 0.01, respectively). More importantly, nuclear PGC-1α content was also significantly decreased (*p* < 0.01). AIT increased nuclear PGC-1α levels and Tfam expression (*p* < 0.05).

## 3. Discussion

The present study demonstrated that MI impaired mitochondrial respiratory functions and reduced ETC activities. These changes are likely to be secondary to pathologically remodeled dynamics witnessed by decreased fusion (mfn2 and OPA1) and increased fission (DRP1). In contrast, eight weeks of AIT attenuated mitochondrial dysfunction, which was evidenced by protected network dynamics. AIT-induced inactivation of ERK1/2-JNK-P53 signaling and increase in nuclear PGC-1α may be key mechanisms accounted for normalized dynamic network. This study comprehensively explored the effects of AIT on myocardial mitochondrial dynamics and functional performance in post-MI hearts.

In our study, we determined that mitochondrial dynamics were pathologically remodeled after MI, as evidenced by decreased expression of mitochondrial fusion proteins mfn2 and OPA1 and increased expression of fission indicator DRP1 (Figure 5), which is primarily attributable to dysfunctional mitochondria. Proper fusion events, which require mfn1/2 and OPA1, and proper fission events, which rely on DRP1, are indispensable for mitochondrial integrity, ETC activities, coupling, oxidative phosphorylation and optimal metabolic output in muscle [9,29]. Disruption of the homeostasis between fusion and fission are usually associated with various diseases, including cardiovascular disease and diabetes [30–32]. However, mfn1/2 and OPA1 overexpression may improve mitochondrial functions and inhibit cardiac failure [33]. Among cardiovascular diseases, studies on mitochondrial network dynamics largely focus on the terminal events of cardiovascular disease, such as heart failure [34], and less on the ischemic myocardium. We have determined that MI disrupted the balance between fusion and fission, indicating that dynamics remodeling may be a common occurrence present throughout the process of MI-induced CHF. Regulatory mechanisms of dynamic remodeling have been unclear until recently, when one research group documented that mfn1/2 expression was noticeably decreased after PGC-1α depletion [35], suggesting that PGC-1α may be a key regulator [36]. Importantly, PGC-1α is also a key regulator of mitochondrial fission [37,38]. In addition to PGC-1α, the oxidative response was involved in the course of mitochondrial fission, but the downstream mechanisms remain elusive. In the current study, our results (Figure 6) indicated that MI induced activated JNK/ERK-P53 signaling, which may represent a novel understanding of pathological mitochondrial fission.

In contrast to the sedentary MI animals, rats subjected to exercise training exhibited improved respiratory function (Figures 3 and 4) and normalized dynamics (Figure 5). However, the protective mechanisms of exercise on mitochondrial dynamics in the failing heart are still unknown. Here, our results indicated that increased antioxidant effects and increased nuclear PGC-1α expression may play essential roles. Various mediators and pathways are involved in exercise-delivered antioxidants in the pathologically remodeled heart. Lower antioxidase levels and injured mitochondrial DNA accompany the oxidative response [39]. Exercise increases mitochondrial [40] and cytoplasmic antioxidase [41,42], which could be beneficial for mitochondrial function and dynamics. Furthermore, endurance training increases vagus nerve activity impaired in CHF [43]. We previously observed that acetylcholine (a vagus nerve neurotransmitter) may attenuate the oxidative response in H9c2 cells [44], indicating that vagus nerve activation may also inhibit the dynamics remodeling.

Along with antioxidant effects, altered PGC-1α (total protein level) is an indicative regulator of mitochondrial dynamic network [45]; however, this concept was challenged by freshly emerging evidence, demonstrating that the state of PGC-1α was chiefly determined by its subcellular location [25] and post-translational modifications [46]. In the present study, the nuclear PGC-1α levels were assessed. The results demonstrated that nuclear PGC-1α levels were decreased after MI (Figure 7). On the basis of previous results (decreased total PGC-1α levels) [47], our results extended the assessment of MI affecting PGC-1α (decreased nuclear content) (Figure 7). PGC-1α is prone to various cellular responses and is involved in various physiological processes; therefore, altered PGC-1α is observed in numerous diseases [48]. In our model, decreased nuclear PGC-1α levels may be detrimental to the stable mitochondrial pool [28] and oxidative phosphorylation subunits [36], thus inducing mitochondrial dysfunction.

AIT increased nuclear PGC-1α expression. The adaptive response of PGC-1α to AIT has been highlighted in previous investigations on metabolic syndrome patients [18] and healthy individuals [17], showing increased PGC-1α mRNA or protein levels. However, the underlying mechanism is still unknown. Disruption of Ca^2+^ circling and decreased Ca^2+^-calmodulin dependent kinase (CaMK) can decrease PGC-1α expression. AIT reportedly normalizes Ca^2+^ handling and increases CaMK expression [24], which could exert salutary effects on PGC-1α. Furthermore, decreased expression of the sirtuin deacetylases was found in experimental models of CHF, which may inactivate mitochondrial ETC and suppress PGC-1α through acetylation [49]. Endurance training reportedly increases sirtuin expression [50], which contributes not only to PGC-1α activity through deacetylation at the post-transcriptional level but also to the enhanced mitochondrial ETC activities. Moreover, our laboratory recently indicated that phosphatidylinositol-3 kinase (PI3K)-protein kinase B (PKB/Akt) signaling likely regulates PGC-1α in mitochondrial nutrient-induced biogenesis [51]. Endurance training activates this signaling cascade [52], implying that the PI3K-Akt signaling cascade may play a key role in exercise-mediated PGC-1α increase. However, the specific mechanism by which AIT increases PGC-1α nuclear localization remains undetermined, and more research is warranted. In addition, the P13K/Akt signaling cascade activates endothelial nitric oxide synthase, which is the key regulator of nitric oxide (NO) [53]. There is emerging evidence that physiological levels of NO could inhibit the opening of the mitochondrial permeability transition pore [54], prevent oxidative stress [55] and boost mitochondrial biogenesis [56]. Furthermore, a recent study documented that NO inhibited DRP-1-mediated mitochondrial fission [57], which is critical for maintaining mitochondrial function. Therefore, PI3K-Akt signaling pathway activation and increased NO release after exercise training could provide comprehensive protection for the mitochondria.

In addition, it is important to note that 3 animals died after surgery upon starting the intervention in the AIT group and only 1 died in the MI alone group. Although the reason for this difference remains unknown, the higher exercise intensities involved in AIT may have resulted in additional acute mortality risks. Consequently, before these results could be translated to humans, further research would be needed to ensure the adaptive health benefits outweighed any acute adverse outcomes. In practice, medical supervision and monitoring would be indispensable when initiating AIT, and an individual’s specific AIT protocol should be determined from the results of a comprehensive physical examination to determine the appropriate intensity, frequency and duration of this intervention.

## 4. Experimental Section

### 4.1. Animals

All of the experimental procedures were performed in strict accordance with the Guidelines on the Care and Use of Laboratory Animals as issued by the Chinese Council on Animal Research and Guidelines of Animal Care. The present study was approved by the Ethical Committee of Xi’an Jiaotong University (Xi’an, Shaanxi, China). 100 male adult Sprague-Dawley (SD) rats (8 weeks old, 180–220 g) were purchased from the Experimental Animal Center of Xi’an Jiaotong University (Xi’an, Shaanxi, China). Rats were individually housed in a temperature-controlled animal room (20–23 °C) under circadian conditions with free access to food and water.

### 4.2. In Vivo Myocardial Infarction Model

After one week of adaptive feeding, 70 of 100 animals were randomly selected for a myocardial infarction operation. The rats were anesthetized by intraperitoneal pentobarbital sodium injection (35 mg/kg). Mechanical ventilation was accomplished with a breath mask linked to a ventilator. The operation was conducted by ligation of the left anterior descending coronary artery (LAD), as previously described [53]. In brief, the rats’ respiratory functions were preserved through use of a ventilator, and body temperature was maintained with an incubator that was fixed to a laboratory bench. After the thoracotomies, a 5.0 silk suture was used to permanently ligate the LAD localized in 3 mm below the left atrium. Wounds were sutured with 5.0 silk sutures that had been treated with iodophor to prevent infection. An electrocardiogram was recorded with an electrocardiograph (Power Lab/4SP; AD Instruments, Sydney, NSW, Australia); change of ST-segment (elevation) was indicator of a successful operation. Sutures for rats in the Sham group were attached without tying.

### 4.3. Experimental Grouping and Animal Use

After MI, each animal was allowed one week for recovery before experimental grouping. In total, six rats died during the MI operation, and one died the day after MI. After one week of natural rehabilitation, the operated animals were designated for evaluation of exercise capacity via adaptive exercise training (15 m/min, 10 min/day, 3 day/week), and those rats that demonstrated better exercise capacity were chosen for AIT. The rats with poor exercise performance were excluded and moved into the sedentary MI group. Thus, all live rats were divided into three groups: sham-operated control (Sham), sedentary MI (MI) and aerobic interval training (MI + AIT). In the sedentary MI group, one rat died in the second week after MI operation. In the AIT group, two rats died in the first week, and one animal died in the third week of AIT. The final number of animals in each group was 30. When the experiment was completed, six rats were used for HE staining, 10 were designated for evaluation of mitochondrial respiratory function, and the other animals were used to assess protein expression by Western blot in each group.

### 4.4. Treadmill AIT Protocol

Animal AIT performed with a treadmill (JD-PT, Jide, Shanghai, China). The AIT protocol consisted of a 10 min warm-up period (50%–55% maximal oxygen uptake (*V*O_2max_)), seven interval training periods (4 min intervals at 80%–90% *V*O_2max_ interspersed with 3 min periods of 65%–75% *V*O_2max_) and a 1 min cool down period [58]. This protocol was performed for 1 h/day, 5 day/week in the morning from Monday to Friday for eight weeks. To adjust the practical running speed and keep a relative training load, the *V*O_2max_ of the AIT animals was evaluated at the beginning of every week during treadmill training, and the AIT procedure was conducted as previously described [59].

### 4.5. HE Staining

HE staining was performed in accordance with a previous method from our laboratory [53]. In brief, excised hearts were first placed in a petri dish filled with 0–4 °C saline and were then fixed with 4% paraformaldehyde for at least 24 h. After dehydrating in alcohol, xylene, baptist wax and paraffin embedding, the heart was cut into 5 μm sections with a microtome, HM500 OM (Walldorf, Germany). Next, after dewaxing and rehydration, the sections were stained with hematoxylin and eosin solutions and were mounted using a coverslip and neutral gum. Image-Pro Plus (Media Cybernetics, Silver Spring, MD, USA) software for Windows was used for picture assembly.

### 4.6. Myocardial Mitochondria Preparations

The tissue around the infarcted areas was used for mitochondrial isolation. Briefly, excised hearts were cut into pieces. The connective tissue was removed with surgical scissors, and residual blood was washed away with medium I (0.25 mol/L sucrose, 3.0 mmol/L HEPES, 0.5 mmol/L EDTA, pH 7.4). Next, samples were deferentially centrifuged in medium I (20 mL, 1200× *g*, 2 min; 50 mL, 1200× *g*, 2 min; 600× *g*, 5 min). The resulting supernatant was homogenized again (10,000× *g*, 10 min). After centrifugation, 5 mL medium II (0.25 mol/L sucrose, 2.0 mmol/L HEPES, 0.5 mmol/L EDTA, pH 7.4) was added to the harvested precipitation, which was homogenized with a Teflon manual homogenizer. The samples were then re-homogenized (10,000× *g*, 10 min). The collected precipitate contained mitochondria. The procedures listed above were performed on ice. The mitochondrial protein concentration was determined using a BCA™ Protein Assay Kit (Pierce 23225; Rockford, IL, USA), with bovine serum albumin as a standard, at room temperature.

### 4.7. Mitochondrial Enzyme Activity Assay

All ETC complexes in frozen mitochondria were detected as described in previous studies performed in our laboratory [60,61]. NADH-CoQ oxidoreductase in complex I was assayed by recording the decrease in absorbing 2,6-dichlorophenolindophenol (DCPIP) at 600 nm in test buffer containing 50 mM Tris-HCl, 0.1% BSA, 1 μM antimycin A, 0.2 mM NaN_3_ and 0.05 mm coenzyme Q1 at pH 8.1. Succinate-CoQ oxidoreductase in Complex II, CoQ-cytochrome C reductase in Complex III, cytochrome C oxidase in Complex IV and oligomycin-sensitive Mg^2+^-ATPase in Complex V activities were also assessed.

### 4.8. Mitochondrial Respiratory Activity Determination

Mitochondrial respiration performance was evaluated using a Clark oxygen electrode (model YSI 5331, Yellow Springs, OH, USA), as previously described [62]. In brief, the reaction was performed in a 3 mL closed thermostatic glass chamber with a magnetic stirrer and respiration medium (pH 7.4) composed of 130 mM KCl, 3.0 mM HEPES, 0.5 mM EDTA, 2.0 mM KH_2_PO_4_, 1 mg/mL BSA, and 2 mg mitochondrial protein. Mitochondrial function was measured at 25 °C. After a 3-min equilibration, mitochondrial respiration was initiated by adding 1 mM glutamate and 0.1 mM malate (final concentration). State 3 respiration began after adding 200 nM ADP. When an evident corner emerged, respiratory function entered into State 4. The RCR is expressed as the respiratory values ratio of State 3 to State 4. The P/O was used for assaying mitochondrial capacity of energy synthesis, and was evaluated by the ratio of phosphorylated ADPs to consumed oxygen atoms during State 3.

### 4.9. Measuring Mitochondrial Membrane Potential

Mitochondrial membrane potential (Δψm) was measured using a Mitochondrial Membrane Potential Detection Kit (Beyotime, No. C2006, Haimen, China). Briefly, membrane potential was monitored by determining the relative amount of dual emissions from mitochondrial JC-1 monomers or aggregates using an Olympus fluorescent microscope (Olympus, Tokyo, Japan) under Argon-ion 520 nm laser excitation. Mitochondrial depolarization was expressed as the increased ratio between green and red fluorescence intensities.

### 4.10. Nuclear Protein Extraction

Nuclear protein was extracted using a commercial nuclear protein and cytoplasmic protein extraction kit P0028 (Beyotime, Haimen, China) in accordance with the manufacturer’s instructions. Briefly, 1 mM tissue homogenate (60 mg/200 μL) was transferred into a centrifuge tube and ice bath for 15 min at 4 °C. The homogenate was then centrifuged at 1500× *g* for 5 min at 4 °C. PMSF protein extraction reagent A (60 mg/200 μL) was added to the collected precipitate, and the sample was vortexed for 5 s. The sample was added to 10 μL protein extraction reagent B and was centrifuged (12,000–16,000× *g*, 5 min, 4 °C). The precipitate was then added to 50 μL nuclear protein extraction reagent and vortexed for 15–30 s per 1–2 min for 30 min. Finally, samples were centrifuged at 12,000–16,000× *g* for 10 min at 4 °C. The harvested precipitate contained nuclear protein. Protein concentrations were calculated using a BCA Protein Assay Kit (Pierce 23225; Rockford, IL, USA).

### 4.11. Western Blot Analysis

Protein expression was assessed by Western blot. The lysate homogenized the shredded samples, which were then centrifuged (13,000× *g*, 15 min, 4 °C). The supernatants were collected, and protein concentrations were calculated using a BCA Protein Assay kit (Pierce, No. 23225; Rockford, IL, USA). Proteins (20–25 μg protein per lane) were separated by 8%–10% SDS-PAGE and transferred to a PVDF membrane using a semi-dry blot-transferring apparatus (Amersham Biosciences, 44601; Buckingshire, UK). Membranes were then blocked with 5% non-fat milk buffer for 1 h at room temperature. The membranes were incubated with anti-PGC1α, anti-Tfam, anti-cytochrome C, anti-DRP1, anti-mfn1, anti-mfn2, anti-OPA1, anti-CS, anti-JNK, anti-phospho-JNK, anti-p53 (all at 1:1000), (Santa Cruz Biotechnology, Santa Cruz, CA, USA); anti-Histone H1 (1:1000), (Santa Cruz Biotechnology, Santa Cruz, CA, USA); anti-GAPDH (1:10,000), (Sigma, St. Louis, MO, USA); and anti-ERK1/2 and anti-Phospho-ERK1/2 (1:1000), (Millipore, Billerica, MA, USA) antibodies overnight at 4 °C. The membranes were then incubated with the corresponding secondary antibodies for 1 h at room temperature. The blots were imagined with ECL-plus reagent, and the optical band density was quantified with Quantity One densitometry software (Bio-Rad, Hercules, CA, USA). Protein expressions were calculated as the gray scale ratios of protein/GAPDH, which were further compared with the Sham group. The results are expressed as the value (%) relative to the Sham group.

### 4.12. Statistical Analysis

The data are presented as the mean ± SEM. Statistical comparisons among groups were performed by one-way analysis of variance (ANOVA) followed by *post-hoc* comparison via Tukey’s test in PASW SPSS 18.0 software for Windows (IBM, Armonk, NY, USA). *p* < 0.05 was considered significant. GraphPad Software Version 5.01 was used for graphing the results (GraphPad Software, La Jolla, CA, USA).

## 5. Conclusions

The chief finding of the present study was that AIT effectively attenuated or even reversed MI-induced mitochondrial injury in the ischemic myocardium, as evidenced by conservation of functional performance and dynamics proteins. Our findings of post-MI-associated mitochondrial dysfunction and the mechanisms by which AIT promotes mitochondrial adaptations may aid in discerning a new therapeutic regimen for MI. The results of the present study indicate that AIT is a non-pharmacological and effective modality for treating individuals with, or who are at risk for, MI and its associated implications.
